# Understanding and managing HIV infection risk among men who have sex with men in rural Uganda: a qualitative study

**DOI:** 10.1186/s12889-021-11365-9

**Published:** 2021-07-04

**Authors:** Lydia Jacenta Nakiganda, Stephen Bell, Andrew E. Grulich, David Serwadda, Rosette Nakubulwa, Isobel Mary Poynten, Benjamin R. Bavinton

**Affiliations:** 1 Kirby Institute, UNSW Sydney, Kensington, NSW 2052 Australia; 2 UQ Poche Centre for Indigenous Health, The University of Queensland, Brisbane, Australia; 3 School of Public Health, The University of Queensland, Brisbane, Australia; 4 Centre for Social Research in Health, UNSW Sydney, Kensington, Australia; 5 Rakai Health Sciences Program, Kalisizo, Uganda; 6 Makerere University School of Public Health, Kampala, Uganda

**Keywords:** HIV, Men who have sex with men, Uganda, Risk reduction, Qualitative

## Abstract

**Background:**

Same-sex sexual relations are criminalised in Uganda, and men who have sex with men (MSM) experience a high burden of HIV infection. In Uganda, health promotion policies focus on equity in healthcare and creating enabling environments. At present there is limited evidence upon which to enhance engagement of MSM in rural settings into effective HIV prevention. To fill this gap, our study explored MSM’s understandings of HIV risk and strategies used to reduce HIV risk in their sexual lives.

**Methods:**

In-depth interviews were conducted with sixteen MSM in rural communities in Southwestern Uganda. Inductive thematic analysis examined men’s perceptions of HIV risk and strategies of reducing their own HIV risks.

**Results:**

Understandings of HIV risk and risk practices were framed by lack of access to condoms, challenges negotiating condom and pre-exposure prophylaxis (PrEP) use, and condomless sex being reported as more pleasurable than sex with condoms. Strategies men perceived as enabling them to manage HIV risk included: PrEP use; condom use; knowing partners’ HIV status; avoiding partners associated with HIV risk; oral sex; withdrawal before ejaculation and washing one’s penis after sex. There were several misconceptions arising from poor HIV prevention knowledge. Strategies reliant on communication and negotiation with sexual partners were inhibited by gendered powered imbalances.

**Conclusions:**

Our findings illustrate that MSM in rural settings in Uganda are making concerted efforts to implement strategies that might reduce risk of HIV transmission and infection within their sexual relationships. Key HIV health promotion and service-related strategies to support MSM with these efforts include an effective condom and lubricant supply chain; a PrEP program in trusted local health units, implemented via discreet community-outreach mechanisms; and same-sex specific HIV-related health promotion.

## Introduction

‘Key populations’ – populations that are most at risk of acquiring or transmitting HIV and thus key to effective HIV responses [1] – are disproportionately affected by HIV and remain underserved in international HIV responses [2, 3]. Men who have sex with men (MSM) is a key population category that refers to all men who engage in sexual and/or romantic relations with other men [4]. This category encompasses a large variety of settings and contexts in which male-to-male sex occurs and the diverse interpretations of ‘men’ and ‘sex’ in societies and cultures internationally [4]. MSM account for 17% of new HIV infections worldwide and globally are 22 times more likely to acquire HIV compared to all other adult men [5]. In sub-Saharan African settings, the estimated HIV prevalence in this population is 17.9% [6].

In Uganda, MSM are a heterogenous and stigmatised population. Same-sex practices are criminalised under sections 140, 141 and 143 of the Penal Code Act, with a maximum sentence of life imprisonment [7]. National HIV policy guidelines and plans identify MSM as a key population due to high rates of new HIV infections but low levels of engagement with HIV testing and treatment services [8–11]. In Uganda’s capital city, Kampala, HIV prevalence among MSM has been estimated at 13.7% [12], increasing to 22.4% among men aged 25 years or older [13]. Available behavioural survey data – also limited to studies in Kampala [14, 15] – document practices that enhance risk of HIV acquisition among MSM, including receptive anal intercourse without condoms, sex with multiple or casual partners, and involvement in selling sex. However, there is limited survey data that enhances understanding about perceptions of risk, risk practices and strategies to reduce risk among MSM in rural Ugandan settings.

The latest Ugandan national HIV strategy identifies a specific need for provision of HIV prevention and health promotion programs – including condom promotion, access to HIV services, biomedical prevention such as pre-exposure prophylaxis (PrEP) and health education – for key populations including MSM [16]. Despite documentation of HIV risks [13, 14, 17] and national guidelines prioritising HIV prevention to MSM [8–10], legal and social contexts constrain the conduct of qualitative research to inform the design of essential person-centred HIV prevention responses within this marginalised population.

Qualitative research about HIV in MSM populations in Uganda has primarily focussed on access to HIV testing, treatment and care services, from the perspectives of health providers [18–20] and MSM [21, 22]. To our knowledge, only two qualitative studies have examined sexually related HIV risk behaviours among MSM in Uganda [17, 23]. These studies – undertaken with MSM in Kampala [17] and 11 other districts across Uganda [23] – report barriers to condom use. However, to date, there is little understanding about risk perceptions and practices among MSM in more remote settings, and no qualitative research has documented the actions Ugandan MSM take to manage HIV risk in their sexual interactions. To address this knowledge gap, drawing on qualitative data collected from MSM in a rural setting in Southwestern Uganda, this paper aimed at understanding MSM’s HIV infection risks and strategies used to reduce HIV risk in their sexual lives.

## Methods

### Study design and setting

We conducted a qualitative descriptive study using in-depth interviews with sixteen men living in eight villages in a district in Southwestern Uganda, located approximately 200 km from the capital city, Kampala. The district is unnamed to protect the identity of study participants. In-depth interviews were used to examine the understandings and actions associated with HIV risk and risk reduction of MSM living in rural communities. We adopted a constructivist approach – an ontological position asserting that social phenomena and their meanings are continually produced, understood and revised through social interactions within specific socio-cultural settings [24].

### Sample and recruitment

Over a period of four months, participants were recruited purposively through peer leaders. Peer leaders are trained MSM volunteers that inform and motivate other MSM to adopt and sustain healthy sexual practices. Peer leaders already known to the research team identified potential study participants, introduced the study, and then shared the lead author’s (LJN) contact details with those who wanted to explore involvement in the study. To ensure access to other MSM through trusted and safe networks, snowball sampling [25] techniques were also used whereby initial study participants introduced the lead author to other potential participants. Prior to commencing data collection, LJN held one-on-one meetings with each participant at the participant’s preferred location to discuss the purpose of the study and associated risks involved in study participation. Participants were given three days to consider participation in the study, which was confirmed by a telephone call to LJN. Inclusion criteria for participants were: male gender identity; being aged 18 years or older; reporting at least one instance of sex with another man in the preceding three months; residing in the specific district where the study took place; and being willing to participate in the study.

### Data collection

All in-depth interviews followed a semi-structured interview guide to elicit participants’ understandings and experiences of sex with other men, risky sexual practices and events, health seeking attitudes and practices, and access to and use of PrEP. The main questions used to stimulate in-depth conversation with interviewees explored: the range of perceived and self-reported sexual risk practices; available HIV prevention activities; HIV prevention strategies adopted by men; experiences engaging with HIV prevention, care and treatment services; and influences that enhanced or inhibited engagement with HIV services. Interviews were conducted in audio-private locations and at a time chosen by participants in order to ensure confidentiality and their own safety. Interviews were conducted by LJN (in English) and RN (in Luganda and English), lasted 45–90 min, and were digitally audio-recorded.

### Data analysis

In advance of data analysis, all interviews were transcribed verbatim and, where necessary, translated from Luganda to English by RN. The transcripts were deidentified, uploaded into NVivo (version 12) and analysed thematically by LJN using open and axial coding techniques [26]. Familiarisation of data occurred through a detailed reading of transcripts, during which LJN made notes to develop an initial inductive coding framework based on data contained in the transcripts. Open coding of transcripts including identifying segments of text that related to specific themes found frequently in the data. Axial coding consisted of identifying relationships between the open codes to create thematic categories. Two main themes were identified – ‘understanding HIV risk’ and ‘managing HIV risk’ – and these structure the findings section. ‘Memos’ [26] were used during all stages of analysis to refine and document a deeper understanding on how codes changed and related with one another.

For each quotation, we provide the ID number, and self-reported sexual positioning identity. Men self-classified their sexual positions into three mutually exclusive categories: those who identified as ‘tops’ exclusively had anal intercourse in the insertive position; ‘bottoms’ exclusively had sex in the receptive position; and ‘versatile’ men engaged in either or both roles.

### Ethics approval and consent to participate

All methods were performed in accordance with relevant guidelines and regulations and was approved by appropriate Ethics committees. Ethical approval was obtained from the Research and Ethics Committee of the Uganda Virus Research Institute (GC/127/19/07/721), the Ugandan Council of Science and Technology (S5128), and the UNSW Human Research Ethics Committee (HC190313). Given the legal status of same-sex relations in Uganda, written consent was not obtained in order to protect study participants from potential risks. Verbal consent was approved by the ethics committees (Ethics Committee of the Uganda Virus Research Institute, the Ugandan Council of Science and Technology, and the UNSW Human Research Ethics Committee) and was recorded as part of the interview. Participants were compensated for their time and transport costs were covered.

## Results

Overall, 16 MSM participated in this study. Participant characteristics are outlined in Table 1.

**Table 1:** Participants profile

Participant characteristics	Number of participants
**Age**	
19–24 years	10
25–30 years	4
30+ years	2
**Education level**	
Primary	5
Secondary	7
University diploma/degree	4
**Occupation**	
Student	2
Employed	9
Unemployed	5
**Sexual position preference by type**	
Insertive (“top”)	8
Receptive (“bottom”)	5
Both (“versatile”)	3
**Sexual orientation**	
Gay/homosexual	7
Bisexual	9
Straight/heterosexual	0
**Experiences with HIV Care Cascade**	
Never tested	6
Tested (last 6 months)	11
HIV positive on treatment	2
On Pre exposure prophylaxis	3
**Perceived HIV risk reduction strategies**	
Condom use (consistent; reported in last 6 months)	10 (2; 8)
PrEP	3
Knowing your partner’s HIV status	5
Avoiding partners associated with HIV risk	3
Oral sex	3
Withdrawal before ejaculation	2
Washing penis after sex	1

### Understanding HIV risk

#### Sex without a condom

Predominant narratives about HIV risk related to men’s practices of condomless sex. Discussions related to the pleasure of sex without a condom, as well as challenges accessing condoms and practices of negotiating condom use with sexual partners.

Participants reported a variety of reasons why condoms were not pleasurable to use. One participant said that condoms “reduced spontaneity” *(ID01, bottom)*, and another explained, “it’s not natural with condoms, it feels like you are masturbating” *(ID05, bottom).* Other participants described difficulties maintaining an erection when wearing a condom and having to remove the condoms in order to ejaculate. Men reported that it was embarrassing to regularly lose an erection during sex:

*I cannot stay hard for a long time if I use a condom… it is awkward. Sometimes you do not know what to do…it’s bad. Those things [condoms] soak your skin, become sticky and cold. You cannot have a happy ending. (ID10, top)*

Participants described having anal sex without condoms as pleasurable and enjoyable, because it was more sensitive, intimate, and spontaneous.

*I do not feel him with a condom. It is like eating sweets wrapped in a polythene. A sweet tastes differently if eaten when it is still wrapped. It is more enjoyable if eaten when unwrapped. It is not pleasurable with a condom. I feel it myself, more sensitive. It is not comparable to sex with a condom. (ID06, versatile)*

Condoms caused discomfort and pain for some participants. Some talked about the pain experienced during sex with a condom as it tends to get dry after a few minutes into sex. Others reported that rough or fast sex brought about “bruises*” (ID13, top),* “pain” *(ID03, bottom)*, “bleeding” or “tears*” (ID15, versatile)* within the anus.

*We do not have lubricants, so it tends to get dry after a few minutes. Ouch… it was very painful. He was so rough on me; he did not listen to me even after my pleas. Everywhere inside was bruised. I had wounds. It is painful sometimes. (ID03, bottom)*.

Several participants reported a lack of knowledge about where to obtain condoms locally and another participant reported not knowing how to use them.

*I hear about condoms, my friends talk… at school they talk… but I have neither seen them nor used them. I do not know where they are sold. I have never used any, I do not know how to use it. (ID13, top)*

Among participants who did know about condoms, some explained that condoms distributed at hospitals were “poor quality, smelly” *(ID13, top)* or “very small” *(ID15, versatile)*. These condoms were not perceived as durable during sex that was rough or took place without silicone- or water-based lubricants, which were reported as not readily available.

*We have a problem we experience as people who act as bottom. We do not have lubricants. There are some lubricants we got but they itch that we cannot use them. They sometimes burn. So, because we cannot find lubricants, we use soft jelly or soap. (ID14, bottom)*

Participants expressed fears of ill-fitting, low quality condoms perforating, tearing, bursting, or getting stuck in the anus. If such incidents were to occur, the men would be required to visit a health unit to access care; this would entail having to reveal their sexual identity in a context where homosexuality is illegal.

*These condoms do not fit… they get stuck inside the anus. I am scared to use them because if it happens, where do you run to? We are not accepted here. It could go up to the stomach and cause death. I would rather not use. After all, HIV is treatable. (ID07, top)*

Some of the participants who identified as bottoms reported challenges influencing the negotiation of condom use with sexual partners. It was reported that only insertive partners had the power to decide whether condoms would be used, as they were perceived as being more masculine.

*I am the bottom… like a woman. I cannot choose to use or reject a condom. That belongs to the top. I have to respect the man in this. (ID16, bottom)*

*My partner decides whether to use a condom or not. I do not say anything because am the bottom. (ID05, bottom)*

#### Challenges to PrEP use

Twelve participants were aware of PrEP and that it was available at some health units. However, among participants who were aware of PrEP, those not on PrEP described several problems impacting their interest in using it or capacity to access it easily. Participants reported issues with PrEP, including that “swallowing that pill daily is problematic” and that PrEP “brings nausea and discomfort” *(ID04, top*), as well as PrEP being costly due to transport fares for refills *(ID16, bottom)*. Others reported potential PrEP-related stigma, or the fear as being perceived to be HIV-positive, as barriers.

*I do not want to be known as someone who is promiscuous, you lose trust with your partner. That medicine is for people who are promiscuous…, I am not, so why swallow it?” (ID12, top)*

*Let me wait until I get started on ARVs…because I will take my drugs every day. Daily dosing even before I am infected with HIV…I cannot use it. People will think am HIV positive. (ID02, bottom)*

### Managing HIV risk

#### Use of condoms

In this sample, two participants used condoms consistently and eight men reported use of condoms at least once in the past six months. Condoms were used to reduce exposure to HIV, sometimes in combination with other strategies like HIV testing. Condom use was common among men with established trusted social networks associated with non-governmental organisations (i.e. with peer leaders) or clinics (i.e. health workers). In these networks, they were regularly reminded to use condoms by health workers and peer leaders.

*I regularly use condoms to protect myself. I do not play sex without using condoms. I mind so much about my health and I cannot have sex without using condoms. I am the top, so I protect my partner as well. (ID04, top)*

*[Non-government organisation] advises us to use condoms consistently. Each time we are going to have sex I use condoms unless if we test and we are both HIV negative. (ID06, versatile)*

#### Use of PrEP

Three participants in this sample reported consistent use of PrEP as a risk reduction strategy. In these men, PrEP was perceived as easy to take and provided reassurance that they were safe from HIV. PrEP was accessed for free at health services alongside regular monitoring of HIV status.

*We get these pills at [clinic] if you want to use. It is free of charge though we do so many medical tests before you start. It is given every month or three months. Before you get your refills, they do more tests. It is good medicine because you know you are protected from HIV. (ID14, bottom)*

Participants on PrEP indicated a good relationship with health workers. In some instances, health workers provided PrEP at convenient community-based locations.

*I take PrEP every 7pm and I have never missed taking PrEP. [Clinic worker] from [clinic] rides here on a motorcycle monthly for my PrEP refills. I prevent myself from getting HIV since one of my partners is HIV positive. (ID01, bottom)*

#### Knowing your partner’s HIV status

Five men described trying to restrict sex to partners whose HIV status they knew. This was common among men who did not like using condoms as an HIV preventive measure. Some used HIV status to inform their sexual decision making, and actively encouraged men who did not know their status to attend HIV testing services before having sex.

*I do not trust condoms so much. That is why I regularly test for HIV. I like having sex with a person whose HIV status I know. I want to know your HIV status first before having sex. I will ask your status first. If you have not tested, I will direct you to [organisation] for a test before we can do it. (ID05, bottom)*

*I do test for HIV regularly. I do not have sex with a person until I know their HIV status. We may not do it immediately at the time until I know, I must protect myself. (ID08, versatile)*

#### Avoiding partners associated with HIV risk

Three men reported deliberate strategies to avoid having sex with someone perceived as being ‘risky’ *(ID15, versatile).* Some participants tried to avoid sexual partners who were known to frequent HIV ‘hot spot’ areas – that is, specific locations associated with elevated HIV burden and transmission risk.

*I try not to have partners from certain areas where almost everyone has HIV. Partners from [hot spot area] are dangerous. Not only do they have HIV but STIs. They give you ‘enziku’ [gonorrhoea] every time. (ID08, versatile)*

Within their sexual networks, other participants reported screening out sexual partners that were perceived to be “dangerous” ones *(ID15, versatile).* One participant explained that this included men who had “more than five concurrent partners”, were “using drugs” or who regularly “tested positive for enziku [*gonorrhoea*]” (*ID08, versatile)*. Another man said:

*I know some of them [risky partners], very promiscuous… smoke weed… want to do it with everyone. When you ask them to test [for HIV], they refuse and instead want to give you money for sex. I want to protect myself. I cannot do it with them, I don’t want to catch HIV. (ID09, top)*

#### Specific sexual practices

Three men discussed practicing oral sex – instead of anal intercourse – to reduce risk of HIV transmission. Men reported a “low or small” *(ID14, bottom)* risk associated with swallowing the ejaculatory fluid rather than ejaculating during anal intercourse. Another participant mentioned “rinsing one’s mouth immediately” (*ID06, versatile*) after oral sex as a safe sex technique.

Other perceived risk reduction techniques used by participants included “washing my penis immediately after sex” *(ID15, versatile)* or the practice of withdrawal before ejaculation with their partners.

*Pulling out before ejaculation helps… because everything is disposed of outside. I do that with my wife too. I know it is the time, I pull out… you do not catch diseases like HIV or this common gonorrhoea from your partner. (ID09, top)*

## Discussion

Our findings provide novel insight about the diverse strategies that MSM in a rural setting used – on their own, in interpersonal relationships and with support from health professionals – to reduce risk of sexual transmission of HIV, despite them living in a social and legal context where same-sex sexual practices are criminalised and heavily stigmatised. These included personal strategies, such as use of PrEP, avoiding partners associated with HIV risk, and washing their penis after sex. Interpersonal strategies – reliant on some form of interaction with their sexual partner – included condom use, withdrawal before ejaculation, knowing their partners’ HIV status, and having oral rather than anal intercourse (not all of these strategies actually reduce risk of HIV transmission) and the innovative institutional health support of outreach services for an increase in free HIV testing and AIDS treatment access. For instance, the most commonly reported risk reduction strategy used by MSM in this study was condoms, which was discussed more by men connected to social networks that incorporated peer leaders (supported by non-governmental organisation) and health workers, who promoted HIV testing and condom use. Such findings support calls in latest international HIV testing and treatment guidelines with a focus on key populations [4, 27] to draw on the existing strengths of community-based mechanisms of HIV support for MSM in Uganda.

Men’s narratives also depicted an array of barriers that intersect to inhibit effective HIV risk reduction. These included interpersonal influences such as condom misconceptions and difficulties negotiating condom use due to gendered norms associated with sexual positioning identities that create power imbalances in sexual decision-making. Institutional influences were associated with health service delivery and the provision of sexual health promotion. These included limited access to free, high quality condoms; a lack of access to appropriate lubricants to enhance comfort and safety of condom use during anal sex; and minimal community-based health promotion outreach work to clarify myths and misinformation. Socio-legal influences inhibited men from seeking support from health services for sexual health problems due to the risks of stigmatisation, discrimination, violence and incarceration associated with disclosing their sexual practices in a context where same-sex sexual relations are criminalised. By documenting such interpersonal, institutional and structural barriers, our findings build substantially on those from studies in Uganda [23] and other sub-Saharan countries [28–33], that point to a lack of knowledge, information and service access as key barriers to HIV risk reduction among MSM.

Sexual pleasure is an important component of sexual health [34] and this resonated strongly through men’s narratives of HIV risk and risk reduction. Condoms were not used by some participants because condomless sex was experienced as more pleasurable, by men engaging in both insertive and receptive intercourse. On the other hand, some men engaging in receptive anal intercourse narrated strong experiences of pain and, on occasion, violence during sexual interactions, over which they had little control or ability to negotiate and change, due to the gendered roles described in this sample.

Some of the men’s attempts to reduce HIV risk – such as oral sex, withdrawal, and condom use or negotiating the partner’s use of condoms – were reliant on the ability to communicate and negotiate with sexual partners. However, such communication was inhibited by gendered norms. Our findings are similar to the influences of power imbalances in MSM sexual decision-making on HIV risk that have been documented in South Africa [35, 36]. In our study, power dynamics reflected heteronormative gender roles enacted in broader Ugandan society, in that the more masculine partner (‘top’) was perceived to have a greater degree of control over sexual decisions than those typically taking the receptive role (‘bottom’). For example, bottoms felt that they could not enforce condom use and expressed concerns about the potential for sexual and non-sexual violence including their inability to decline condomless sex. The influence of masculinity on condom decision-making and sexual positioning suggests masculinity is a significant influence on effective HIV risk management among MSM. Our findings suggest further research on the role of masculinity on practices that increase HIV risk among MSM.

Our findings revealed misconceptions arising from limited same-sex sexual health promotion in this rural setting. This is perhaps not surprising, due to the criminalised status of same-sex sexual relations, which is likely to hinder HIV-related health promotion and support. Limited understanding of HIV risk among some MSM populations has been documented in Uganda [23], Kenya [37] and Tanzania [38], particularly in relation to HIV transmission via anal intercourse and condom use. However, our analysis revealed specific gaps in HIV transmission knowledge that inhibited men’s ability to identify appropriate risk reduction strategies. Examples included perceptions that HIV risk was only associated with vaginal sex, and that withdrawing before ejaculation was a protective practice.

MSM’s narratives about PrEP illustrate the exciting possibilities for biomedical prevention among men in rural settings. Men in this study were aware of the protective effects of PrEP against HIV acquisition. These findings align with other PrEP studies conducted in Uganda [39], Zimbabwe [40], Nigeria [41], and Kenya [42] showing high awareness of PrEP in MSM, and willingness to take daily PrEP. Important barriers to PrEP use mentioned by men in this sample included distance to designated clinics, low HIV risk perceptions, HIV-related stigma arising from assumptions made by others about someone being seen taking daily pills and perceived side effects of taking the daily pill. Nevertheless, there is potential for considerable uptake of PrEP among MSM in this setting. Our data show that health worker and peer driven outreach are models perceived by PrEP users as important in increasing PrEP access and uptake in this population.

### Limitations

Our study has some limitations. First, this exploratory study consisted of a small sample size (i.e. 16 participants). This was a result of recruitment challenges associated with participants’ worries of the legal consequences that may arise if their sexuality became public knowledge. The second issue relates to issues associated with the way men were recruited into the study. While all participants were recruited from remote rural areas, some of the men were strongly associated with non-governmental networks that provided them with health information and enabled access to health services. As a result, the issues raised by these men may differ from other MSM who are not connected to such support networks. Third, despite efforts to recruit a wide range of men, the majority were aged between 18 and 30 years. This was because of their greater willingness to participate in the interviews than the older MSM in these communities. As a result, the findings reported in this paper may not reflect the feelings and experiences of older generations of MSM in this rural setting. Despite these limitations associated with the sampling and recruitment of men involved, our study has provided new insight into MSM’s perceptions of HIV risk, and strategies men used to manage HIV risk in intimate relationships, from a population that is hard to engage in research due to legal policies that criminalise and stigmatize their sexual practices.

### Implications for policy and practice

This study provides useful information on potential avenues to enhance HIV risk reduction practices among MSM in a rural Ugandan setting. Our analysis points to several practical lessons regarding HIV programs to support MSM in such settings. First, an effective condom and lubricant supply chain that ensures condoms are accessible, are of high quality and are widely distributed with appropriate health promotion about how to use both, is vital for pleasurable safe sex and for HIV prevention among MSM in this setting. Second, establishing a PrEP program in trusted local health units and implemented via trusted, discreet community-outreach mechanisms would enable HIV risk reduction among MSM. Third, same-sex specific HIV-related health promotion, outreach and communication are essential to support existing risk reduction efforts and ensure men employ genuine safe sex practices. Such efforts should be framed in latest best practice in comprehensive sexuality and sexual health education, which include a focus on sexual pleasure as well as sexual negotiation and communication skills. These will need to recognise and find ways to address the highly gendered power dynamics at play in these men’s sexual relationships. Finally, our data illustrate that for MSM to engage effectively with health services, they must feel safe enough within the context of trusted relationships with healthcare workers, to seek the care they need. Given the legal risks associated with same-sex sexual relationships, underpinning all HIV strategies with MSM requires the delivery of health promotion and HIV services in safe spaces, and within social networks that include peers and health service providers to guarantee trusted, approachable healthcare services.

## Conclusion

This study documents the diverse personal, interpersonal and institutional strategies and support mechanisms that influenced HIV risk reduction among MSM in a rural setting in Southwestern Uganda. Support through peer networks and health worker outreach strategies encouraged and enabled use of condoms and PrEP for some participants. However, gendered power dynamics inhibited safe sex negotiation and communication within intimate relationships, and socio-legal and policy constraints restricted men’s access to sexual health care in safe clinical settings. To support MSM to have safe, pleasurable sex lives in rural settings such as this, we recommend a multifaceted approach that includes effective condom and lubricant supply chains; PrEP programs that draw on trusted health provider community-outreach strategies; same-sex specific comprehensive sexuality and sexual health promotion education; and ongoing efforts to create safe spaces in clinical and community settings based around trusted patient-provider health care relationships and peer networks.

## Data Availability

Restrictions apply to the availability of study data, and so this data is not publicly available due to individual privacy concerns and the safety of study participants because of the illegality of same sexual relations. However, the data that support the findings of this study are available from the corresponding author on special request.
